# Why and how would we implement a lung cancer screening program?

**DOI:** 10.1186/s40985-015-0010-3

**Published:** 2015-11-05

**Authors:** Idris Guessous, Jacques Cornuz

**Affiliations:** 1 Unit of Population Epidemiology, Division of primary care medicine, Department of Community Medicine, Primary Care and Emergency Medicine, Geneva University Hospitals, Geneva, Switzerland; 2 Division of Chronic Diseases, Institute of Social and Preventive Medicine, Lausanne University Hospital, University of Lausanne, Lausanne, Switzerland; 3 Department of Epidemiology, Rollins School of Public Health, Emory University, Atlanta, GA USA; 4 Department of Ambulatory Care and Community Medicine University of Lausanne, Lausanne, Switzerland

**Keywords:** Lung cancer, Screening, Low dose computed tomography, Overdiagnosis, Smoking, Evidence, Implementation

## Abstract

For decades, lung cancer has been the most common cancer in terms of both incidence and mortality. There has been very little improvement in the prognosis of lung cancer. Early treatment following early diagnosis is considered to have potential for development. The National Lung Screening Trial (NLST), a large, well-designed randomized controlled trial, evaluated low-dose computed tomography (LDCT) as a screening tool for lung cancer. Compared with chest X-ray, annual LDCT screening reduced death from lung cancer and overall mortality by 20 and 6.7 %, respectively, in high-risk people aged 55–74 years. Several smaller trials of LDCT screening are under way, but none are sufficiently powered to detect a 20 % reduction in lung cancer death. Thus, it is very unlikely that the NLST results will be replicated. In addition, the NLST raises several issues related to screening, such as the high false-positive rate, overdiagnosis and cost. Healthcare providers and systems are now left with the question of whether the available findings should be translated into practice. We present the main reasons for implementing lung cancer screening in high-risk adults and discuss the main issues related to lung cancer screening. We stress the importance of eligibility criteria, smoking cessation programs, primary care physicians, and informed-decision making should lung cancer screening be implemented. Seven years ago, we were waiting for the results of trials. Such evidence is now available. Similar to almost all other cancer screens, uncertainties exist and persist even after recent scientific efforts and data. We believe that by staying within the characteristics of the original trial and appropriately sharing the evidence as well as the uncertainties, it is reasonable to implement a LDCT lung cancer screening program for smokers and former smokers.

## Introduction

In 2007, we discussed the situation and perspective of lung cancer screening [1]. At that time, we presented the high mortality of lung cancer, described the promising screening modalities, and characterized the ongoing and planned trials. Overall, we concluded that until the completion of these trials, widespread lung cancer screening intervention should be avoided. Eight years and a large randomized clinical trial (RCT) later, we propose to discuss why and how we would implement a lung cancer screening program, if any. In this narrative review, we briefly review the current evidence regarding the influence of lung cancer screening on lung cancer mortality as well as the major issues and limitations related to lung cancer screening. We then discuss the different factors that should be considered when designing and implementing a lung cancer screening program.

### Why would we implement a lung cancer screening program?

Several reasons can motivate the implementation of a lung cancer screening program. These reasons include the decade-long burden of lung cancer, the lack of meaningful improvement in lung cancer prognosis, the identification of a well-defined population at high risk of lung cancer, and the evidence from a large, well-designed RCT.

#### Burden of lung cancer

Lung cancer represents a huge public health burden. Worldwide, it is the leading cause of death from cancer, with 1.6 million deaths reported each year [2]. It affects both males and females, in which it is either the first or second (respectively) leading cause of death from cancer [3]. In fact, lung cancer causes more deaths than do colorectal, breast, and prostate cancer combined [1].

A remarkable observation that contrasts with other frequent cancers in adults is that the 5-year survival rate of lung cancer remains –in 2015— very low. In developed countries, the overall 5-year survival rate is 20 % or less [4]. This is because lung cancer is generally diagnosed at late stages, when treatments do not improve the prognosis. The poor chance of cure at late stages of lung cancer contrasts with the 80 % 5-year survival rate observed when treatment is initiated at an early stage [5].

**Fig. 1:**
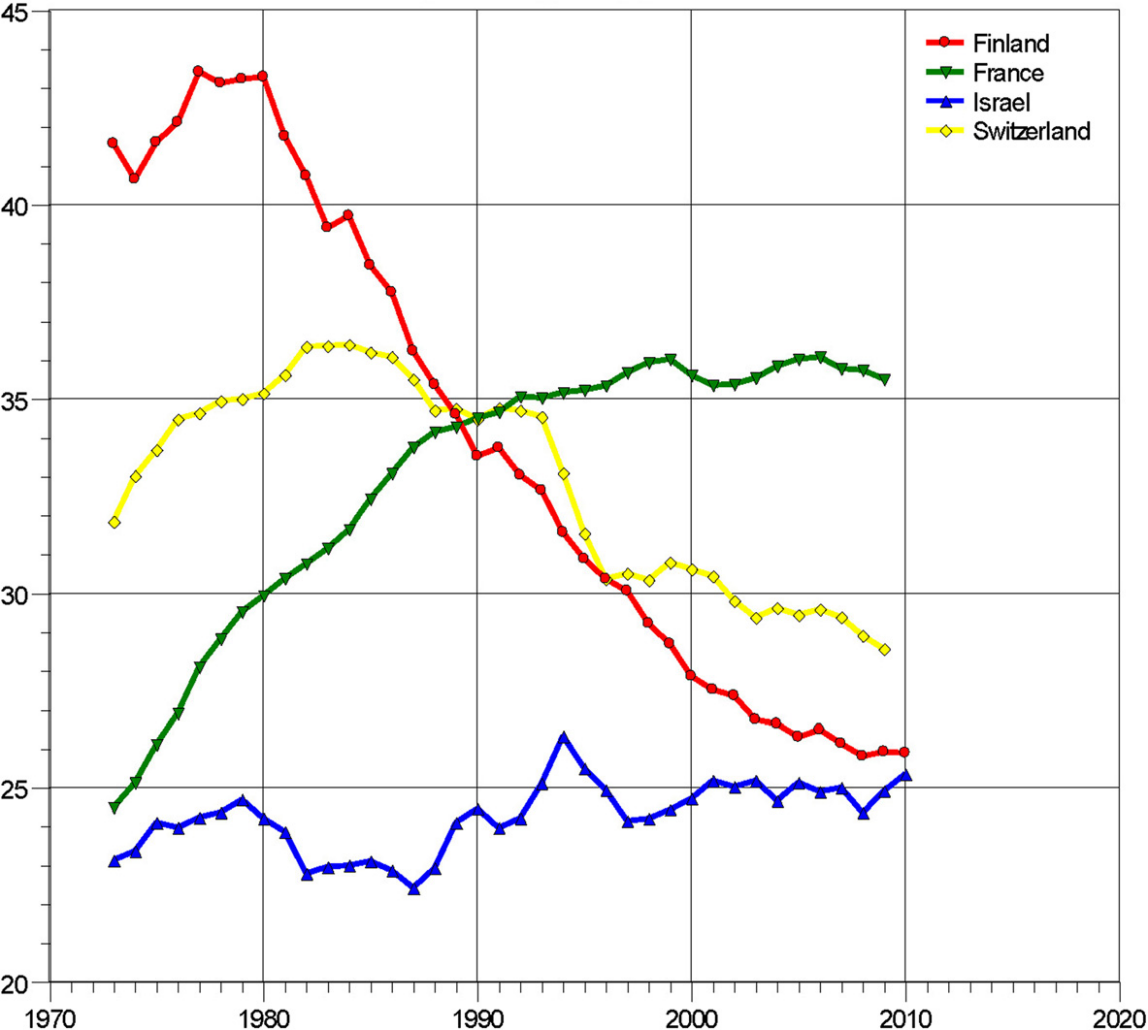
Lung cancer mortality in selected countries in the European region 1970–2014 (data from the Health for All Data Base, World Health Organization, European Region, 2014, courtesy of TH Tulchinsky). Footnote: SDR, standardized death rate

Although mortality trends differ between countries, even within the European region (Fig. 1), this grim situation has existed for decades, and novel treatment modalities that could improve the 5-year survival rate of lung cancer are not well established [6].

**Fig. 2:**
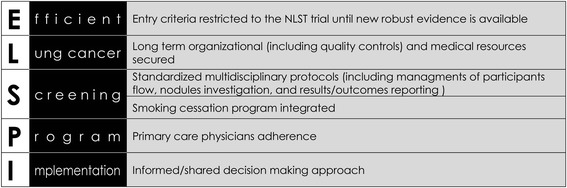
Proposed outline for implementing an efficient, low-dose computed tomography lung cancer screening program

#### A well-defined population at risk

Screening is considered legitimate in the presence of a frequent disease that is generally diagnosed too late for treatment to be curative [7, 8]. Screening might be planned when a population at risk has been clearly identified. Smoking is the primary cause of lung cancer, and population variation in lung cancer incidence and mortality clearly reflects smoking behavior [9]. Compared with never smokers, smokers have a 20-fold increased risk of dying from lung cancer [10]. There is a decreasing but persistent risk among former smokers, at least within the 15 years after quitting [11].

#### Evidence regarding lung cancer screening intervention

Until recently, there was no recommended screening strategy for lung cancer, reflecting the negative results of trials showing no mortality reduction following screening programs using chest X-ray and sputum examination (for a review see [1]). The Prostate, Lung, Colorectal, and Ovarian Cancer Screening (PLCO) trial was the first large RCT conducted to determine the effectiveness of annual lung cancer screening by chest X-ray [12]. It was conducted in the US and randomized 154,900 participants aged 55–74 years to annual chest X-ray screening or usual care for 4 years with 13 years of follow-up. Annual chest X-ray screening did not reduce lung cancer mortality compared with usual care [5, 12].

The development of fast, high-resolution computed tomography (CT) scans allows the acquisition of multiple slice images within a single breath hold by the patient. With multiple images, the 3-dimensional shape of small nodules can be characterized [13]. Diagnostic CT scan examination uses 200 milliampere seconds (mAs), but a lower dose (approximately 60 mAs) can be used for screening purposes. Imaging with this low-dose CT (LDCT) is of lower quality than with full dose CT, but it is better than images provided by chest X-ray. The first evaluations of LDCT as a screening tool were done in observational (*i.e.*, non-experimental) studies, and they have been summarized previously [1]. Because of their potential biases, observational studies are of limited use when trying to determine the effectiveness of lung cancer screening (and more generally when trying to determine the effectiveness of one treatment or test over another). RCTs –the supreme paradigm for epidemiological research—are needed [14]. Several LDCT lung cancer screening RCTs have been conducted or are ongoing, but the National Lung Screening Trial (NLST) [15] was and will likely remain the largest RCT to examine the effectiveness of such screening at reducing death from lung cancer [16]. The NLST, the main results of which were published in 2011 and in subsequent analyses in 2013, is the largest and most expensive (>$200 million) RCT conducted in the US for a single cancer screen [17]. The main characteristics and results of the NLST are presented in Table 1 and briefly discussed below.

**Table 1:** Main characteristics and results of the National Lung Screening Trial (NLST), 33 sites in the United States

	Intervention arm	Control arm
Screening test	Low dose computed tomography	Chest X-ray
Entry criteria		
Health status	Asymptomatic	
Age, years	55 to 74	
Smoking status	≥30 pack-years or had been smokers within the previous 15 years	
Screening interval	Annually	
Screening duration	3 years	
Number of participants randomized	26,722	26,732
Male, N (%)	15,770 (59.0)	15,762 (59.0)
Number of participants aged ≥65 years (%)	7,110 (26.6)	7,110 (26.6)
Number of former smokers (%)	13,860(51.9)	13,832 (51.7)
Number of current smokers (%)	12,862 (48.1)	12,900 (48.3)
Year 1 (baseline)		
Number screened	26,309	26,035
Positive results (%)	7191 (27.3)	2387 (9.2)
Complete diagnostic follow-up (%)	7049 (98.0)	2348 (98.3)
Thoracotomy (%)	197 (2.8)	96 (4.1)
Had lung cancer (%)	270 (3.8)	136 (5.7)
Year 2		
Number screened	24,715	24,089
Positive results (%)	6901 (27.9)	1482 (6.2)
Complete diagnostic follow-up (%)	6740 (97.7)	1456 (98.2)
Thoracotomy (%)	148 (2.2)	44 (3.0)
Had lung cancer (%)	168 (2.4)	65 (4.4)
Year 3		
Number screened	24,102	23,346
Positive results (%)	4054 (16.8)	1174 (16.8)
Complete diagnostic follow-up (%)	3913 (96.5)	1149 (97.9)
Thoracotomy (%)	164 (4.2)	44 (3.8)
Had lung cancer (%)	211 (5.2)	78 (6.6)
Overall (Year 1–3)		
Number of screening tests	75,126	73,470
Positive results (%)	18,146 (16.8)	5043(16.8)
Complete diagnostic follow-up (%)	17,702 (97.5)	4953 (98.2)
Thoracotomy (%)	509 (2.9)	184 (3.7)
Had lung cancer (%)	649 (3.6)	279 (5.5)
Death within 60 days after most invasive diagnositc procedure	10	11
Person-years	144,103	143,368
Lung cancer death	356	443
Rate per 100,000 person-years	247/100,000	309/100,000
Overall death	1877	1998
Rate per 100,000 person-years	1302 /100,000	1394/100,000

From August 2002 through April 2004, 53,454 participants were randomly assigned to annual LDCT lung cancer screening scans versus chest X-ray for three consecutive years in 33 different sites. The eligible participants were high-risk people, defined as adults aged 55–74 years who had smoked at least 30 pack years or former smokers who had quit 15 years ago or less. The NLST participants appeared to be younger, more educated, and more frequently former smokers than the comparable US eligible population [18, 19]. Adherence to screening was very high (93 %) in both arms [20]. In the case of positive findings (*i.e.*, LDCT arm: at least one noncalcified nodule ≥4 mm in longest diameter or other abnormality suspicious for lung cancer; and chest X-ray arm: any noncalcified nodule or mass), decisions about how to proceed were left to the referring physician [2].

The incidence of lung cancer was 645 cases per 100 000 person years (1060 cancers) in the LDCT group compared with 572 cases per 100 000 person years (941 cancers) in the chest X-ray arm. In the LDCT screening arm, 356 deaths from lung cancer occurred (247 per 100 000 person-years) compared with 443 deaths (309 per 100 000 person-years) in the chest X-ray arm. After a 6.5-year median follow-up, the trial found that lung cancer mortality was reduced from 1.7 % in the chest X-ray arm to 1.4 % in the LDCT arm, a statistically significant relative risk reduction of 20 % (95 % CI, 6.8 to 26.7 %). The trial also showed a statistically significant relative reduction of 6.7 % (95 % CI, 1.2 to 13.6 %) in overall mortality [15]. LDCT screening translated to three or four fewer lung cancer deaths per 1000 participants (*i.e.*, an absolute risk reduction of lung cancer death by three to four per 1,000 individuals screened) [21]. The number needed to screen to prevent 1 lung cancer death was 320 persons undergoing three annual LDCTs. In comparison, screening mammography estimates suggest that 465 to 601 women must be screened to save one life [19].

Other (mostly European) published and ongoing LDCT lung cancer screening RCTs (*e.g.*, Dante; MILD; DLCST; NELSON; Italung; Depiscan; Lusi; UKLS) have been presented and discussed elsewhere [2, 20, 22]. Spiro SG and Navani N [6] all of the trials are smaller than the NLST and insufficiently powered to detect a 20 % risk reduction. The results from pooled analyses will of course be interesting to better define who should be screened, for how long, and how frequently [20], but even when combined, these trials will likely not have sufficient statistical power to change the conclusions drawn from the NLST [16].

Like others, we believe that the NLST provides good (but not replicated) evidence that LDCT lung cancer screening reduces not only lung cancer mortality but also overall mortality. Aside from smoking cessation, which reduces the risk of lung cancer mortality by 50 %, LDCT lung cancer screening is the most efficient intervention to reduce lung cancer mortality among high-risk individuals. Following several organizations (*e.g.*, US Preventive Services Task Force [USPSTF], American Cancer Society [ACS]), we would recommend LDCT lung cancer screening to eligible adults. However, like many others [23], we are aware of several issues and questions that the NLST has raised or has left unanswered, including the false positive rate, overdiagnosis, eligibility criteria, and cost.

#### False-positive rate

More than any other cancer screen, LDCT lung cancer screening generates false positives (*i.e.*, participants who had a positive scan but were not found to have lung cancer). In the NLST trial, the mean false-positive rate was approximately 28 % (range 3.8–69.0 %, depending on the trial site) [15]. A systematic review of RCTs and cohort studies found that 20 % of LDCT screening tests lead to false positive findings [24, 25]. This of course suggests that LDCT screening tests have low specificity, but a crucial point is to assess the impact of such a high rate of false positives both for the participants and for the screening program.

In the NLST, some of the positive tests were not considered to require follow-up, but 13 % of the participants underwent further clinical testing. Among the false positives, some had bronchoscopy and others underwent needle biopsy. Of the approximately 26,000 participants in the LDCT arm, 16 participants, ten of whom had lung cancer, died within 60 days after an invasive diagnostic procedure (0.59 %) [15]. Overall the incidence of at least one complication was only 1.4 % in the LDCT arm. Among participants who did not have cancer, <0.1 % of the positive screening tests led to a major complication after an invasive procedure [15]. As discussed by Detterbeck *et al.* [26], it is important to recognize that some deaths may be unrelated events that happened to occur after a screening procedure. For instance, in NLST, some deaths were presumably unrelated, as 1.9 and 1.5 per 10,000 occurred within 60 days, respectively, in the LDCT arm and in the chest X-ray arm when the diagnostic evaluation involved only an imaging evaluation [26].

These data demonstrate that the risk of major complications from LDCT screening exists but is very low. Thus, the benefits of LDCT screening seem to outweigh the risk of lung cancer death in the absence of LDCT screening, though a false-positive result may be associated with short-term emotional distress that is reversible over time [27].

Yet another related issue is the increased risk associated with radiation exposure. Ionizing radiation causes DNA breaks that might lead to cancer. An analysis of data from 15 countries has estimated that up to 3 % of cancer diagnosed to the age of 75 years may be attributable to diagnostic X-rays, including CT scans [28]. However, the assumptions were subject to considerable uncertainty, and different societies of radiology have rated the additional lifetime risk for fatal cancer from LDCT as very low [5]. It is estimated that one death from cancer per 2500 people screened may be caused by radiation from three LDCT screens plus related diagnostic imaging [24]. Given that LDCT screening resulted in one lung cancer death avoided per 320 persons screened, the benefits of screening outweigh the risks from radiation exposure.

#### Overdiagnosis

Overdiagnosis in cancer screening is defined as a cancer that does not evolve or even diminishes or a cancer that progresses so slowly that the patient dies from other causes without ever having developed cancer symptoms. Ruano-Ravina *et al*. [29] as stressed by Ruano-Ravina *et al.* “overdiagnosis is not just a possibility, it is a fact.” [29] Grannis FW Jr found little evidence for substantial numbers of overdiagnosed [30], whereas the USPSTF estimated overdiagnosis to occur in 10-12 % of lung cancers [31], and Patz *et al.* used data from the NLST to estimate an overdiagnosis rate with three annual screens of 16-23 % [32].

In an RCT and in the absence of overdiagnosis, once the trial ends, the number of cancer diagnoses should be the same in the intervention and control arms. Persistent excess cancer in the screening arm suggests overdiagnosis [19]. Evidence of overdiagnosis has been observed in a number of trials [33], but no RCT has been long enough, given that it may take 10–15 years for the detection rates in the two arms to equalize [6].

Similar to other cancer screens (*e.g.*, prostate cancer screening), all participants diagnosed with lung cancer are theoretically treated so that the natural history of untreated screening-detected lung cancers cannot be determined. Even RCT design, which is helpful for estimating the magnitude of overdiagnosis, is not immune from overdiagnosis.

#### Cost-effectiveness

Although several cost-effectiveness studies of LDCT lung cancer screening have been published (with a wide range of conclusions) [34–36], only one was based on the NLST data. The cost-effectiveness analysis based on NLST data found $81,000 per quality adjusted life year gained [37]. This is very similar to other cancer screening programs, including mammography and colonoscopy [23, 38, 39]. The eligible population for LDCT lung cancer screening is very large, and the impact of LDCT lung cancer screening on healthcare cost is obviously not trivial. Models concluded that if it were implemented nationally in the US, an LDCT screening program for a population of 18 million adults with a smoking history of at least 30 pack years would lead to an added annual cost of $4.4 billion for the US healthcare system [34]. This is significant, but such a program i) would prevent over 18,000 premature deaths per year just in the US [40], ii) would be cost-effective, and iii) would be the only intervention (with smoking cessation) that provides a meaningful (20 %) decrease in the risk of lung cancer death. Considered together, this should probably motivate the implementation of an LDCT lung cancer screening.

### How would we implement a lung cancer screening program?

Although several experts have stressed that areas of uncertainty exist regarding the benefits and harms of screening the community at large [16, 21, 40], LDCT lung cancer screening programs have started in the US. Some institutional experiences have demonstrated that NSLT findings are generalizable and that translation of the lung cancer screening concept into clinical practice is feasible [41]. A 2013 survey of 19 US sites (selected best hospitals, top cancer and pulmonary disease centers) found that 79 % of the sites had a CT screening program [42], 73 % of the screening programs used the NLST entry criteria, and 93 % included a smoking cessation program. Below, we discuss why an LDCT lung cancer screening program should indeed be based on the NLST entry criteria and why it should include a smoking cessation program.

#### Eligibility criteria

The facts that the eligibility criteria for screening in the NLST apply to <30 % of the lung cancers in the US [43] and that the NLST participants were healthier and better educated than the general US population have often fueled calls to redefine the entry criteria for LDCT lung cancer screening. Lung cancer risk prediction models for determining whom to screen have been proposed (*e.g.*, The PLCO lung cancer risk calculator, which is available online at http://www.brocku.ca/lung-cancer-risk-calculator). Some of these models include information on chronic obstructive pulmonary disease or pulmonary function (*e.g.*, forced expiratory volume in 1 s, FEV1) [44, 45]. Targeting of LDCT lung cancer screening according to risk has been motivated by a secondary analysis of the NLST data showing that the 60 % of participants at highest risk for lung cancer death (based on risk quintiles) accounted for 88 % of the screening-prevented lung cancer deaths, while the 20 % of NLST participants at lowest risk accounted for only 1 % of prevented lung cancer deaths [46]. Another secondary analysis of the NLST data compared the outcomes of screening between Medicare-eligible (aged 65 to 74 years) and younger (aged 55 to 64 years) subgroups of NLST participants in the LDCT group. The authors found that the absolute benefits and harms of screening were greater in the 65 to 74 years group. Because lung cancer incidence and mortality were higher among participants in the older group at baseline, they had more to gain from screening [47].

Although developing such models and conducting these secondary analyses are certainly important to further our understanding of the different impacts of LDCT lung cancer screening, it is worth noting that the strengths of the initial randomization are habitually lost in post hoc analyses. It is therefore very difficult to recommend LDCT lung cancer screening beyond the original NLST trial entry criteria. Age criteria, however, are often discussed. For example, the USPSTF extended the age limit to 80 based on microsimulation modeling by the Cancer Intervention and Surveillance Modeling Network (CISNET) Lung Group [31]. Extending the age is not trivial given that false-positive rates, complication rates from biopsy of pulmonary nodules, postoperative mortality, and competing risk for death all increase with age [48]. The benefit-risk ratio of LDCT screening among adults aged >74 is unknown.

In the absence of additional robust evidence on the benefits of screening when applied to broader populations, we recommend –like others [40]— that programs limit the use of LDCT screening to those individuals who meet the NLST eligibility criteria.

#### Smoking cessation

Smoking is the greatest risk factor for lung cancer, and smoking cessation reduces the risk of dying from lung cancer by more than 50 % [11]. Two major findings have highlighted the need to include a smoking cessation program within a lung cancer screening program (*e.g.*, enrollment in a smoking cessation program at the time of screening). First, screening for lung cancer has been shown to be a teachable moment for smoking cessation [5]. The smoking cessation rates in the LDCT studies were four-fold the rate observed in the general population (4 % vs 16 %) [2]. Although the probability of subsequent smoking seems to be inversely associated with the abnormality of the screening result, both the screening and control arms were more likely to stop smoking compared with the general population [49]. Furthermore, studies have not shown an increased smoking rate in persons with negative screening results, indicating that participants are not using negative findings to continue or resume smoking [5]. Additionally, smoking cessation programs (including pharmacotherapy for nicotine dependence) are very cost-effective interventions [50], with costs of $5000 per quality-adjusted life-year [51]. Although robust evidence of the beneficial effect of including a smoking cessation program in an LDCT lung cancer screening program remain to be gathered, it seems legitimate to enroll smokers in a smoking cessation program at the time of screening. The feasibility of this approach will depend on the prevalence of current smokers, which can be low in certain settings.

Frameworks for efficient implementation of lung cancer screening programs have been published [52, 53]. In addition to suggesting eligibility criteria and the inclusion of smoking cessation programs, the frameworks discuss the screening frequency, screening duration, nodule algorithm, need for longitudinal registries and standards for certification of screening centers. In general, the recommendations follow the characteristics of the NLST trial. We would like to stress two additional factors to be considered when implementing LDCT lung cancer screening (Fig. 2): involving health care providers and using an informed-decision making approach.

#### Health care providers

When cancer screening entry criteria are limited to factors such as age (*e.g.*, colorectal cancer screening) and gender (*e.g.*, breast cancer screening), participants can be invited *via* an existing (non-medical) registry. However, when additional entry criteria such as smoking history are needed, programs often rely on the medical system (medical system-based) to contact patients. Lung cancer screening, if implemented, would mostly work through patients, health care providers including physicians, and, more specifically, primary care physicians. If primary care physicians are not convinced of the efficacy of the program and/or are not fully aware of the screening entry criteria, the lung cancer screening program is unlikely to work. This potential gap between recommendation and implementation is very reminiscent of other medical-system based screens (*e.g.*, abdominal aneurysm screening among smokers and former smokers) [54]. As discussed below (informed-decision making), we believe that primary care physicians do not need to be convinced that the benefits of lung cancer screening outweigh the risks (this mainly concerns the patient), but rather they should be convinced that the program would offer his/her patient the best care possible would he/she decide to participate. Similar to all cancer screening programs, lung cancer screening programs need to be of the highest quality. Medical system-based screening programs such as a lung cancer screening program need to let the primary care physicians know about their high level of quality. Of note, although our discussion is focused on primary care physicians, the involvement of other health care professionals could be more appropriate, depending on the health care system. In fact, a combination of physicians’ referrals, other health professionals’ referrals and even self-referrals could be the most efficient and cheapest means to reach out to the eligible population to improve screening uptake.

#### Informed decision making

If a lung cancer screening program has to be implemented, it *must* utilize an informed decision-making approach. This requirement is *sine qua non* given that LDCT lung cancer screening does both good and harm and presents uncertainties. Surveys have shown that health care providers often discuss the pros of screening but seldom discuss the cons [55]. To better inform patients, guidelines on counseling about the risks and benefits of lung cancer screening prior to screening have been published [26, 56], and decision aids are available [55]. For example, to support primary care physicians in the informed consent process, some programs send each qualified patient a 4-page list of frequently asked questions [41]. Despite the evidence of harm and the uncertainty, frequent screening for disease has been performed for years without systematic informed- and shared-decision making approaches. Informed/shared-decision making was only recently introduced into major health organization screening guidelines [57, 58]. Lung cancer screening has no choice but to be a perfect application of informed choice [58]. Moreover, the evidence-based information should consider the participant’s literacy and ethnicity and should be culturally appropriate.

## Concluding remarks

In addition to smoking cessation, the 20 % decrease in lung cancer deaths reported in the large, well-designed NLST trial represents the greatest progress in lung cancer reduction. In this review, we present the main reasons for implementing LDCT lung cancer screening in high-risk adults and discussed the main issues related to LDCT lung cancer screening. Frameworks for the efficient implementation of lung cancer screening programs are available. We stress the importance of primary care physicians and informed-decision making. In 2007, we were waiting for the results of RCTs. Although the only large trial could not answer all of the questions, evidence is now available. Similar to almost all other cancer screens, uncertainties exist and persist even after colossal efforts such as the NLST. However, we believe that while staying within the characteristics of the original trial and appropriately sharing the evidence as well as the uncertainties, it is reasonable to implement a LDCT lung cancer screening program for smokers and former smokers. Long-lasting resources and quality controls are, of course, prerequisites.
